# Identification of COPD Inflammatory Endotypes Using Repeated Sputum Eosinophil Counts

**DOI:** 10.3390/biomedicines10102611

**Published:** 2022-10-18

**Authors:** Augusta Beech, Natalie Jackson, Dave Singh

**Affiliations:** 1 Division of Immunology, Immunity to Infection and Respiratory Medicine, Faculty of Biology, Manchester Academic Health Science Centre, School of Biological Sciences, Medicine and Health, The University of Manchester, Manchester M23 9LT, UK; 2 Medicines Evaluation Unit, Manchester University NHS Foundation Trust, Manchester M23 9QZ, UK

**Keywords:** COPD, sputum, eosinophil, eosinophil repeatability, endotype, microbiome

## Abstract

Higher blood and sputum eosinophil counts are associated with a greater response to corticosteroids in COPD. Low blood eosinophil counts exhibit greater stability over time whereas higher counts demonstrate more variability. Stability of airway eosinophil levels is less well understood. We have studied the stability of sputum eosinophil counts. Differential cell count data for COPD patients (*n* = 100) were analysed. Subjects with two sputum eosinophil counts, 6 months apart, were included in the analysis. Patients were stratified based on baseline sputum eosinophil count into ‘low’, ‘intermediate’ and ‘high’ groups: eosinophil^LOW^ (<1%), eosinophil^INT^ (1–3%) and eosinophil^HIGH^ (≥3%). Sputum eosinophil counts showed good stability (rho = 0.61, *p* < 0.0001, ICC of 0.77), with 67.4% of eosinophil^LOW^ patients remaining in the same category on repeat sampling. Bland–Altman analysis of the whole cohort (median difference between measurements = 0.00%, 90th percentile = −1.4 and 4.7%) showed greater variation at higher counts. This was confirmed by the wider 90th centiles in the eosinophil^INT^ (−1.50 to 5.65) and eosinophil^HIGH^ groups (−5.33 to 9.80) compared to the eosinophil^LOW^ group (−0.40 to 1.40). The repeatability of sputum eosinophil counts was related to the baseline eosinophil count; sputum eosinophil^LOW^ COPD patients were relatively stable over time, while the eosinophil^HIGH^ group showed greater variability. These results can facilitate the identification of COPD endotypes with differential responses to treatment.

## 1. Introduction

Chronic obstructive pulmonary disease (COPD) is a heterogeneous disease [1]. This causes a high degree of variability between individuals in the clinical responses to pharmacological interventions [2]. Studies performed approximately 20 years ago showed that higher sputum eosinophil counts are associated with greater clinical responses to corticosteroid treatment in COPD patients [3,4]. There is a positive correlation between blood and sputum eosinophil counts [5], and blood eosinophil counts (BEC) have subsequently emerged as an accessible biomarker that can be used in clinical practice to predict the response to inhaled corticosteroids (ICS) in COPD patients at increased exacerbation risk [6]. The relationship between BEC and ICS responses is a continuum, with <100 and >300 eosinophils/µL being estimated thresholds to identify individuals with the lowest and highest probability, respectively, to show a positive clinical response [5,6]. 

Higher blood and sputum eosinophil counts in COPD patients are associated with increased type 2 (T2) inflammation in the lungs [7,8]. Furthermore, several studies have demonstrated that lower blood and sputum eosinophil counts are associated with increased presence of proteobacteria, the phyla encompassing Haemophilus influenzae (*H. influenzae*) and Moraxella catarrhalis (*M. catarrhalis*) [9,10,11]. These associations between eosinophil counts, T2 inflammation and the microbiome appear to be determinants of the clinical response to ICS and the risk of bacterial infection [5,12,13].

Studies of the stability of BEC in COPD over time have shown greater stability at lower eosinophil counts [14,15], with the majority of counts < 100 eosinophils/µL remaining below this threshold, or showing only small changes to move just above this threshold [16,17]. In contrast, there is greater variation at higher BEC. A small study (*n* = 14) reported that lower submucosal eosinophil counts in COPD bronchoscopic biopsies were relatively stable over time, while higher eosinophil counts show greater variation over time, with increased heterogeneity throughout the bronchial tree [18]. Chronic inflammation is a dynamic process, influenced by the interaction between external stimuli and internal homeostatic regulatory mechanisms [19,20]. These blood and bronchial biopsy studies support the concept that the presence of eosinophilic inflammation in COPD is dynamic, showing temporal variation over time [18]. Some studies have focused on defining a subgroup of COPD patients with “persistently high” eosinophil counts, but perhaps this is not a useful definition, as the presence of eosinophil associated inflammation can be expected to be variable. It is probably more useful to identify individuals with “persistently low” eosinophil counts, who are likely not to respond to ICS and have a microbiome with increased risk of *H. influenzae* infection [5,12,13]. 

This study further tests the hypothesis, suggested by previous COPD studies using blood samples [16,17], that a subgroup of individuals have low eosinophil counts that are relatively stable over time [14,15,16,17], while higher eosinophil counts demonstrate more fluctuation [16,17]. The same pattern was previously observed in a small study using COPD bronchial mucosal biopsies [18], but larger studies using lung derived samples are needed to properly understand the temporal variation of eosinophils in the lungs. This study has analysed repeated sputum samples from a COPD cohort (n = 100) to further investigate this issue. 

## 2. Materials and Methods

### 2.1. Subjects

We performed an analysis of repeated sputum cell counts obtained from observational research at our centre. COPD patients who provided baseline and 6 month sputum data were included (*n* = 100). Patients were recruited from the Medicines Evaluation Unit (Manchester University NHS Foundation Trust). Patients were included if they were aged ≥40 years old, met global initiative for chronic obstructive lung disease (GOLD) criteria for the diagnosis of COPD [6] and had a smoking history of ≥10 pack years. Patients were not included if they were using maintenance antibiotics or oral corticosteroids, or had a previous asthma diagnosis. Sputum data from 48 patients in this analysis have been previously reported, although not for the purpose of assessing eosinophil repeatability [9,21]. All patients provided written informed consent using protocols approved by the local Ethics Committees (05/Q1402/41, 10/H1016/25 and 16/NW/0836). 

### 2.2. Study Design

Sputum and blood differential cell counts (DCCs) were obtained during stable state, defined as no symptom defined exacerbation within 4 weeks of sampling. Symptoms were assessed using the modified medical research council questionnaire (mMRC) [22], COPD assessment test (CAT) [23] and health related quality of life using the St George’s Respiratory Questionnaire (SGRQ-C) [24]. Lung function measurements were performed according to guidelines [25,26]. 

### 2.3. Sputum Measurements

Sputum induction was performed, and spontaneous samples were collected where induction was not possible (approximately 2.5% of samples). Briefly, sputum plugs were selected from saliva using forceps and processed using a 2-step method consisting of a Dulbecco’s phosphate-buffered saline (D-PBS) wash step followed by a dithiothreitol (DTT) step and preparation of cytospins for DCC as previously described [27], full details for measurement of DCC are provided in the supplementary material.

### 2.4. qPCR Detection of Common Respiratory Pathogens

The detection of *H. influenza*, *M. catarrhalis*, Streptococcus pneumoniae (*S. pneumoniae*), and Pseudomonas Aeruginosa (*P. aeruginosa*), were analysed according to eosinophil sub-group as previously described [9,21]. Further methodological detail can be found in the online data supplement. 

### 2.5. Blood Measurements

Blood eosinophil measurements were performed by The Doctors Lab (TDL, London, UK) or Wythenshawe Hospital clinical laboratory (Manchester, UK), full details for the measurement of blood DCC are provided in the supplementary material. 

### 2.6. Statistical Analysis

3 groups were identified by stratifying patients based on baseline sputum eosinophil count into ‘low’, ‘intermediate’ and ‘high’ groups: eosinophil^LOW^ (<1%), eosinophil^INT^ (1–3%) and eosinophil^HIGH^ (≥3%) [4,28]. For parametric data, statistical analysis was performed using analysis of variance (ANOVA) with Tukey’s multiple comparison post hoc test. Statistical analysis for non-parametric data was performed using Kruskal–Wallis test followed by post-test analysis using Dunn’s multiple comparison test and spearman’s rank test assessed associations between variables. Comparisons between categorical data were assessed using a chi-squared test. Repeatability was assessed using (1) Bland–Altman analysis; within subject differences for sputum eosinophil percentage retained a non-Gaussian distribution after log transformation, therefore the median difference and the 90th centile were used as descriptive statistics [29] (Prism 8.0, GraphPad, San Diego, CA, USA) and (2) Intraclass correlation coefficient (ICC) analysis was based on an absolute agreement, two-way mixed effects model [30] (SPSS 25.0, IBM, Armonk, NY, USA). ICC values are interpreted as excellent (>0.75), fair to good (0.40–0.75), or poor (<0.40) correlations [29]. For ICC analysis, sputum data were normalised via a Log(x + 1) transformation to account for zero values. *p* < 0.05 was considered statistically significant. 

## 3. Results

The demography and baseline sputum data for the cohort (*n* = 100) is shown in Table 1; the mean post-bronchodilator forced expiratory volume in 1 second (FEV_1_) was 63.4% predicted, with 47.6% of patients having ≥1 exacerbation in the previous 12 months. Mean CAT and SGRQ scores were 19.3 and 47.7 respectively. The proportion of ICS users was 66.0%.

The eosinophil^LOW^, eosinophil^INT^ and eosinophil^HIGH^ groups defined at baseline comprised 43 (43.0%), 35 (35.0%) and 22 (22.0%) subjects respectively. The clinical characteristics of these groups were mostly similar (Supplementary Table S1). Sputum characteristics for the three groups are presented in Table 2; eosinophil counts were highest in the eosinophil^HIGH^ group as expected, and there was a non-significant trend for neutrophil and macrophage cell count x10^6^/g to be lower in the eosinophil^HIGH^ group. 

### 3.1. Repeated Sputum Eosinophils Counts

**Figure 1:**
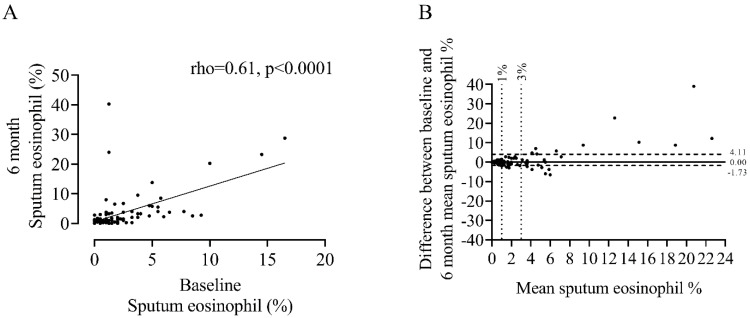
Association between baseline and 6 month measures of sputum eosinophil % (**A**) and Bland–Altman plot of the difference versus the mean of two repeat measurements (**B**). Horizontal solid black line in panel B represents the median difference, horizontal dashed black lines represent the 90th percentile and the vertical dotted lines represent thresholds of 1 and 3% sputum eosinophil indicative of eosinophil^LOW^ and eosinophil^HIGH^ groups respectively. n = 100.

Repeated sputum eosinophil counts showed a positive correlation between baseline and 6 m (rho = 0.61, *p* < 0.0001, Figure 1), with an ICC = 0.77 indicating excellent repeatability between measurements. This association was present in both ICS and ICS non-users (Supplementary Figure S1). Visual inspection of the Bland–Altman plot (Figure 1B) shows a widening trend with greater differences between measurements observed at higher sputum eosinophil counts (median difference = 0.00%, 90th percentile = −1.73 to 4.11%). 

**Figure 2:**
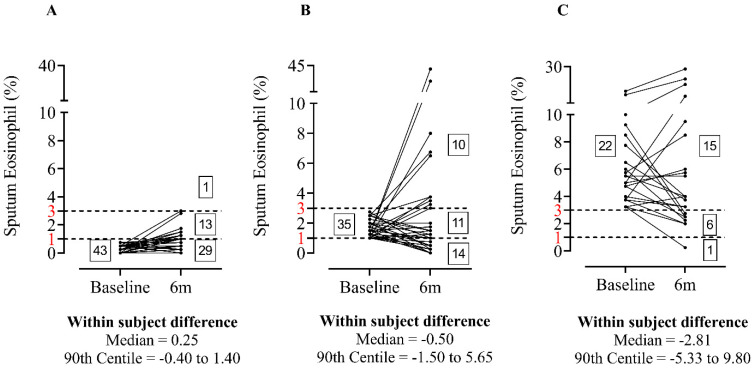
Stability of sputum eosinophils stratified by baseline sputum eosinophil %; Eosinophil^LOW^ (**A**), Eosinophil^INT^ (**B**) and Eosinophil^HIGH^ (**C**), horizontal dashed lines and red numbering represent represent thresholds of 1 and 3% sputum eosinophil indicative of eosinophil^LOW^ and eosinophil^HIGH^ groups respectively. n = 43, 35 and 22 respectively.

The median within-subject difference between repeat visits was lower in the eosinophil^LOW^ group (0.25, 90th centile = −0.40 to 1.40) compared to eosinophil^INT^ (−0.50, 90th centile = −1.50 to 5.65) and eosinophil^HIGH^ groups (−2.81%, 90th centile = −5.33 to 9.80) (Figure 2), although this was not statistically significant (ANOVA = 0.06). The 90th centiles demonstrated greater variation at higher eosinophil counts. 

The majority of eosinophil^LOW^ patients remained in the same category (67.4%) after repeat sampling (Figure 2A). 68.2% of eosinophil^HIGH^ patients remained in the same category (Figure 2C). Only 2 patients moved between the eosinophil^LOW^ and eosinophil^HIGH^ groups. For the eosinophil^INT^ group, less patients remained in the same category (31.4%, Figure 2B). No significant association between change in sputum eosinophil % and FEV_1_ between visits were observed, details can be found in the supplementary material. 

Ninety-six patients had between visit exacerbation data available; 32 patients had ≥1 exacerbation. The correlation and ICC between sputum eosinophil % at baseline and 6 months was similar between subjects with and without exacerbations (rho = 0.67 and 0.64, respectively, *p* < 0.001 for both, ICC = 0.86 and 0.75 respectively; Supplementary Figure S2). 

### 3.2. Sputum Eosinophil Counts and BEC

**Figure 3:**
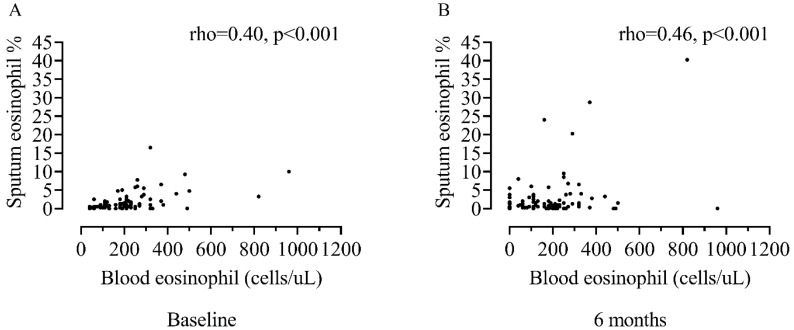
Association between sputum and blood eosinophil counts at baseline (**A**) versus 6 months (**B**). n = 75 and 84 respectively.

Sputum eosinophil counts were correlated with BEC at baseline (rho = 0.40, *p* < 0.001, Figure 3A) and 6 months (rho = 0.46, *p* < 0.001, Figure 3B).

**Figure 4:**
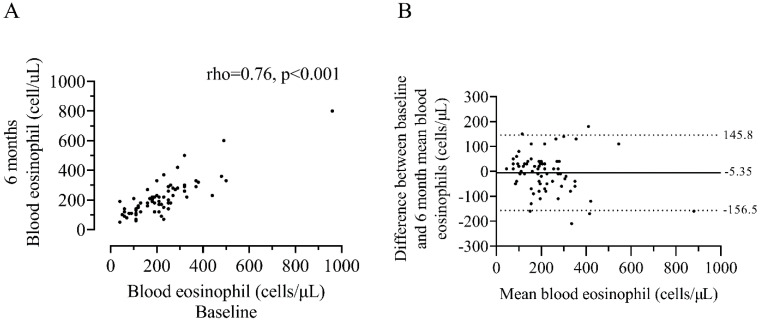
Association between baseline and 6 month measurements of blood eosinophil counts (**A**) and a Bland–Altman plot of the difference versus the mean of two repeat measurements (**B**). Horizontal solid black line in panel B represents the mean difference and horizontal dotted black lines represent the upper and lower 95% limits of agreement. n = 71.

Repeated blood eosinophil counts showed a strong correlation between visits (rho = 0.76, *p* < 0.001, Figure 4) with an ICC of 0.89 indicating excellent repeatability. Bland–Altman analysis demonstrated a mean difference of −5.35 cells/µL with 95% limits of agreement (LOA) between −156.5 and 145.8 (cells/µL). Visual inspection of the plot shown in Figure 4B, similarly to sputum measurements, shows a widening trend, with greater differences between measurements observed at higher mean blood eosinophil counts.

### 3.3. Bacterial Colonisation and Sputum Eosinophil Counts

Thirty-four patients had a sufficient sample for bacterial qPCR at baseline. Bacterial load of *H. influenzae* was significantly higher in the eosinophil^LOW^ group compared to eosinophil^HIGH^ (2.67 × 10^4^ versus 2.75 × 10^2^ genome copies/mL, *p* = 0.03, Supplementary Table S2). Bacterial load for *S. pneumoniae*, *M. catarrhalis* and *P. aeruginosa* were similar between groups. 

## 4. Discussion

In this cohort of 100 COPD patients, the overall repeatability of sputum eosinophil counts at 6 months was excellent (ICC = 0.77). Greater variation was observed at higher sputum eosinophil counts while the eosinophil^LOW^ subgroup was relatively stable over time. These sputum results are compatible with similar previous observations using blood and bronchial biopsy samples from COPD patients [16,17,18]. The results reported here, taken together with these previous COPD eosinophil studies, indicate that blood and lung eosinophil counts can follow distinct patterns over time; (1) persistently low eosinophil counts, signifying low levels of T2 inflammation and possible infection with *H. influenzae* and (2) higher eosinophil counts with greater potential for variation over time.

The overall excellent repeatability of sputum eosinophil % reported here is consistent with repeatability reported for sampling intervals up to 3 months [31,32]. However, the different patterns observed over time can facilitate the identification of different subtypes of COPD; individuals with persistently low eosinophil counts may have a different microbiome and increased risk of infection with proteobacteria, while individuals with higher (and perhaps variable) eosinophil counts may be suitable candidates for pharmacological treatments, including ICS and novel therapies against T2 inflammation. Overall, movement between the eosinophil^LOW^ and eosinophil^HIGH^ subgroups was rare (only 2 out of 100 patients). We suggest that eosinophil^INT^ is not a stable category, but consists of individuals who can be classified as either eosinophil^LOW^ or eosinophil^HIGH^ after further testing. Therefore, repeat sampling of eosinophil^INT^ individuals may help uncover whether an individual belongs to a lower or higher category (eosinophil^LOW^ or eosinophil^HIGH^ respectively).

Wang et al. reported changes in COPD sputum cell profiles and microbiome over a time period up to 2 years, showing that eosinophilic samples (sputum eosinophils ≥ 3%) may become non-eosinophilic (<3%), but rarely changed microbiome to acquire Haemophilus [11]. We showed that the eosinophil^LOW^ group had higher sputum levels of *H. influenzae*, and that movement between the eosinophil^LOW^ and eosinophil^HIGH^ groups was uncommon. Our results are therefore compatible with those of Wang et al., suggesting that the eosinophil^LOW^ and eosinophil^HIGH^ groups are largely independent over time. Our microbiome results are consistent with other studies in different cohorts showing increased infection with proteobacteria such as *H. influenzae* in subjects with low eosinophil counts [10,11,33]. The eosinophil^HIGH^ group, while temporally variable, harbour a more balanced microbiome enriched with several specific non-dominant genera such as *Campylobacter* [11].

Higher blood and sputum eosinophil counts have been associated with higher levels of T2 inflammation in the lungs, notably increased levels of chemokine (C-C motif) ligand (CCL) -24, CCL26, interleukin (IL) -5, IL-13, chloride channel accessory (CLCA)-1 and cystatin SN (CST) -1 [7,8]. Furthermore, it has been reported that eosinophilic COPD patients have higher levels of airway immunoglobulins, and greater anti-bacterial immunity [34]. These relationships between eosinophils, T2 inflammation and the microbiome provide potential mechanistic explanations for differential treatment responses [8,13,35].

The relationship between sputum and blood counts observed here is consistent with many studies that have also shown an associations between these two measurements (literature (reviewed in [5]); the strength of the relationship reported here (rho = 0.40–0.46) aligns within that previously reported within the literature (rho = 0.18–0.70) [13]. There are multiple explanations proposed for the variable strength of association seen in previous studies including inclusion criteria, sample size, the method of reporting (i.e., number of decimal places used) [5] and diurnal variation of blood eosinophil counts [36].

The present analyses regarding the stability of BEC are comparable to previous reports, indicating good-excellent repeatability between repeat measurements, taken 6 months apart (ICC = 0.77 in the present study versus 0.73–0.89 reported elsewhere [17,37]). Furthermore, we observed greater variability at higher BEC, again consistent with previous reports [16,17,38,39].

Limitations of this study include a moderate sample size (n = 100) which became smaller upon subgrouping. Furthermore, longer-term follow up data would be informative, although this is often hampered in COPD cohort studies by dropouts over time, for example due to declining health.

In conclusion, we observed a subgroup of COPD patients (eosinophil^LOW^) with relatively stable sputum eosinophil levels over time, in comparison to eosinophil^HIGH^ patients with considerably greater variability. These results can facilitate the identification of these COPD endotypes who have differential responses to ICS treatment and different respiratory microbiome profiles.

## Figures and Tables

**Table 1:** Baseline Demographics: Summaries are presented as percentages or Mean (SD) as appropriate (n = 100 *).

Characteristic	n = 100
Gender n (Female/Male)	34/66
Age	65.2 (7.5)
Smoking status (Current %)	42.0
Pack years	42.3 (18.9)
BMI (kg/m^2^)	27.8 (5.1)
Exacerbations (1 year period)	0.89 (1.2)
0 (%)	52.4
1 (%)	23.8
≥2 (%)	23.8
Post FEV_1_ (L)	1.8 (0.5)
Post FEV_1_ (%)	63.4 (17.4)
GOLD Category (%)	
1	16.0
2	65.0
3	19.0
4	0.0
mMRC	3.0 [0.0–4.0]
CAT	19.3 (7.4)
SGRQ-C (Total)	47.7 (18.3)
Atopy (%)	10.0
Chronic bronchitis (%)	74.6
ICS Use (%)	66.0
LABA + LAMA + ICS (%)	53.0
LABA + LAMA (%)	7.0
ICS only (%)	2.0
LABA only (%)	1.0
LAMA only (%)	14.0
No inhaled medication (%)	9.0
Sputum characteristics	
Sputum total cell count × 10^6^/g	7.49 [0.62–100.9]
Sputum Neutrophil (%)	73.63 [15.25–99.50]
Sputum Eosinophil (%)	1.00 [0.00–16.50]
Sputum Lymphocyte (%)	0.25 [0.00–4.75]
Sputum Macrophage (%)	18.00 [0.50–79.50]
Sputum Epithelial Cells (%)	2.13 [0.00–5.25]
Sputum Neutrophil cell count × 10^6^/g	4.65 [0.03–98.08]
Sputum Eosinophil cell count × 10^6^/g	0.08 [0.00–2.45]
Sputum Lymphocyte cell count × 10^6^/g	0.01 [0.00–0.64]
Sputum Macrophage cell count × 10^6^/g	1.22 [0.04–10.13]
Sputum Epithelial cell count × 10^6^/g	0.13 [0.00–2.45]

* The following data were missing; 20 retrospective exacerbation history, 10 atopy categorisation, 37 chronic bronchitis categorisation, 25 mMRC questionnaires, 29 CAT questionnaires and 37 SGRQ’s, 2 sputum cell % (except for sputum eosinophil %), 10 total cell counts.

**Table 2:** Baseline sputum characteristics for separate groups defined by baseline eosinophil %; Eosinophil^LOW^ Eosinophil^INT^ and Eosinophil^HIGH^: Summaries are presented as percentages or median [range] as appropriate (n = 100 ^#^).

Baseline Sputum Characteristic	Eosinophil^LOW^ n = 43	Eosinophil^INT^ n = 35	Eosinophil^HIGH^ n = 22	*p*-Value
Sputum total cell count × 10^6^/g	8.53 [0.96–100.9]	9.53 [0.62–58.78]	6.30 [1.43–49.40]	0.27
Sputum Neutrophil (%)	74.50 [15.25–99.50]	77.75 [29.75–97.00]	67.25 [24.25–87.50]	0.13
Sputum Eosinophil (%)	0.25 [0.00–0.75]	1.50 [1.00–2.75] ***	5.25 [3.25–16.50] ***^,+++^	<0.0001
Sputum Lymphocyte (%)	0.00 [0.00–4.75]	0.25 [0.00–2.00]	0.00 [0.00–3.50]	0.79
Sputum Macrophage (%)	18.00 [0.50–79.50]	15.25 [1.25–60.00]	19.50 [1.50–54.00]	0.62
Sputum Epithelial Cells (%)	1.25 [0.00–60.50]	2.13 [0.00–40.50]	2.75 [0.00–16.25]	0.31
Sputum Neutrophil cell count × 10^6^/g	5.15 [0.03–98.08]	6.74 [0.32–57.01]	2.90 [0.35–36.11]	0.11
Sputum Eosinophil cell count × 10^6^/g	0.03 [0.00–0.34]	0.13 [0.00–0.59] ***	0.29 [0.00–2.45] ***	<0.0001
Sputum Lymphocyte cell count × 10^6^/g	0.02 [0.00–0.64]	0.01 [0.00–0.26]	0.00 [0.00–0.21]	0.09
Sputum Macrophage cell count × 10^6^/g	1.25 [0.04–10.13]	1.24 [0.08–3.51]	1.05 [0.10–3.41]	0.54
Sputum Epithelial cell count × 10^6^/g	0.12 [0.00–2.45]	0.16 [0.00–1.95]	0.13 [0.00–0.73]	0.45

^#^ The following data were missing; 2 sputum cell % (except for sputum eosinophil %), 10 absolute sputum inflammatory cell counts. ***, *p* < 0.001 compared to Eos^LOW^. ^+++^, *p* < 0.001 compared to Eos^INT^.

## Data Availability

The datasets generated and/or analysed during the current study are not publicly available.

## Supplementary Materials

The following supporting information can be downloaded at: https://www.mdpi.com/article/10.3390/biomedicines10102611/s1; Figure S1. Association between baseline and 6 month measures of sputum eosinophil % for ICS users (A) versus non-users (B). n = 66 and 34, respectively, Figure S2. Association between baseline and 6 month measures of sputum eosinophil % for those with no exacerbations between visits (A) versus those with ≥1 (B). n = 64 and 32, respectively, Table S1. Baseline demographics for separate groups defined by baseline eosinophil %; Eosinophil^LOW^ Eosinophil^INT^ and Eosinophil^HIGH^, Table S2. Baseline bacterial qPCR results for common respiratory pathogens measured in sputum, for separate groups defined by baseline eosinophil %; Eosinophil^LOW^ Eosinophil^INT^ and Eosinophil^HIGH^, Table S3. Details of qPCR targets and the lower limits of detection for qPCR detection of different PPMs. Table S4. Baseline demographics for separate groups defined by change in eosinophil % between baseline and 6 months using a threshold of 3% sputum eosinophils. References [40,41] are cited in the supplementary materials.
