# Supplementary material for: Identification of COPD Inflammatory Endotypes Using Repeated Sputum Eosinophil Counts
Source: Biomedicines. 2022 Oct 18;10(10):2611. doi: 10.3390/biomedicines10102611 (PMC9599170; doi:10.3390/biomedicines10102611)
Supplement: Supplementary file 1 [file biomedicines-10-02611-s001.zip › biomedicines-1947818-supplementary.pdf]

Online Data Supplement

Stability of sputum eosinophil cell counts in COPD

Augusta Beech[1, 2], Natalie Jackson[2], Dave Singh[1, 2]

[1]Division of Infection, Immunity and Respiratory Medicine, School of Biological Sciences, Faculty of Biology, Medicine and Health, Manchester Academic Health Science Centre, The University of Manchester, Manchester, UK

[2]Medicines Evaluation Unit, Manchester University NHS Foundation Trust, Manchester, UK

Corresponding author:

Augusta Beech

Department of Medicine and Health

University of Manchester

Education and Research Centre, M23 9LT, UK

Tel: +44 (0)161 946 4050 Fax +44 (0)1619461459

Email address: augusta.beech@manchester.ac.uk

Methods

Sputum measurements (evaluation of DCC)

Differential cell counts were estimated manually using cell suspension obtained from a whole sputum sample, mounted onto slides to create cytospins. Leucocyte viability was assessed by staining cells with 0.4% Trypan Blue solution (Merck, UK). Cells were stained using Eosin Y and Methylene Blue (Rapi-diff II stain, Atom scientific, UK). Four hundred of the following non-squamous cells were evaluated based on structure and characteristics; macrophages, neutrophils, eosinophils, lymphocytes and bronchial epithelial cells. Squamous cells were counted but not included in the count of 400 cells. Each sample was objectively assessed for quality; if the leukocyte viability was <50% and/or the squamous cell percentage was >30%, the sample was discarded on the basis of poor quality. DCC slides were quality checked and counts validated by a second observer, if there was a difference in counts of >10% between observers, the average of the two counts were reported.

qPCR Detection of Common Respiratory Pathogens

DNA was extracted from homogenised sputum samples using QIAamp DNA mini Kit (QIAGEN, Crawley, West Sussex, UK); bacterial DNA was stored at −80 °C. Real-time qPCR was performed on *Haemophilus Influenzae* (*H. influenzae*), *Moraxella Catarrhalis* (*M. catarrhalis*), *Streptococcus Pneumoniae* (*S. pneumoniae*) and *Pseudomonas Aeruginosa* (*P. aeruginosa*), targeting the lipo-oligosaccharide glycosyltransferase-encoding gene (lgtC) of *H. influenzae*, the CopB outer membrane protein-encoding gene of *M. catarrhalis*, the autolysin-encoding gene (lytA) of *S. pneumoniae* and the gyrB gene of *P. aeruginosa* as previously described [1,2]. Details of primers and probes can be found in supplementary table 3. The thresholds for defining colonisation with individual bacterial species were based on the upper

limits of a healthy control cohort (details reported elsewhere [3]); *H. influenzae* = 3.22 x 10^5, *M. catarrhalis* = 3.72 x 10^3, S. *pneumonia* = 7.09 x 10^6 and *P. aeruginosa* = 1.68 x 10^2 genome copies / mL.

Blood measurements

Differential cell counts were estimated from EDTA-treated whole blood, using the automated systems; Sysmex XN10 & XN20 analysers (Wythenshawe Hospital clinical laboratory Manchester, UK) and Sysmex XN 9000 & XSi analysers (The Doctors Lab, London, UK), *via* fluorescence flow cytometry.

Results

The clinical characteristics of patients found to be intermittently in the eosinophil^{HIGH} group over 6 months were no different to those persistently in the eosinophil^{HIGH} group or those with a sputum eosinophil % persistently <3% (Table S4).

Change in FEV_1 over 6 months

Mean change between visits for the whole cohort was -0.03L and -1.28% for absolute and % predicted FEV_1 respectively. No association between change in FEV_1, absolute or % predicted, and change in sputum eosinophil % between visits was observed (p = 0.18 and 0.27, respectively). Furthermore, there was no association between change in blood eosinophil count between visits and FEV_1, absolute or % predicted (p=0.75 and 0.98 respectively).

Changes in absolute and % predicted FEV_1 between visits for groups based on sputum eosinophil count at baseline were not different between $Eosinophil^{LOW}$ $Eosinophil^{INT}$ and $Eosinophil^{HIGH}$ groups. Furthermore, no association was observed between change in FEV_1, absolute or % predicted, and change in sputum eosinophil % between visits, for $Eosinophil^{LOW}$, $Eosinophil^{INT}$ and $Eosinophil^{HIGH}$ groups ($p = 0.60$ and 0.32, 0.84 and 0.74, and 0.09 and 0.08 respectively).

Change in ICS use over 6 months

We observed a change in ICS use for 7 / 98 patients, n=5 started ICS and for n=2 ICS treatment was removed (information for n=2 patients was not available). Baseline blood and sputum eosinophil counts, and change in eosinophil counts between visits were no different between those with a change in ICS versus those without. Furthermore, we found no association between change in eosinophil counts and FEV_1 in patients with a change in ICS use. However, the sample size here was too small to infer meaningful conclusions.

Figures

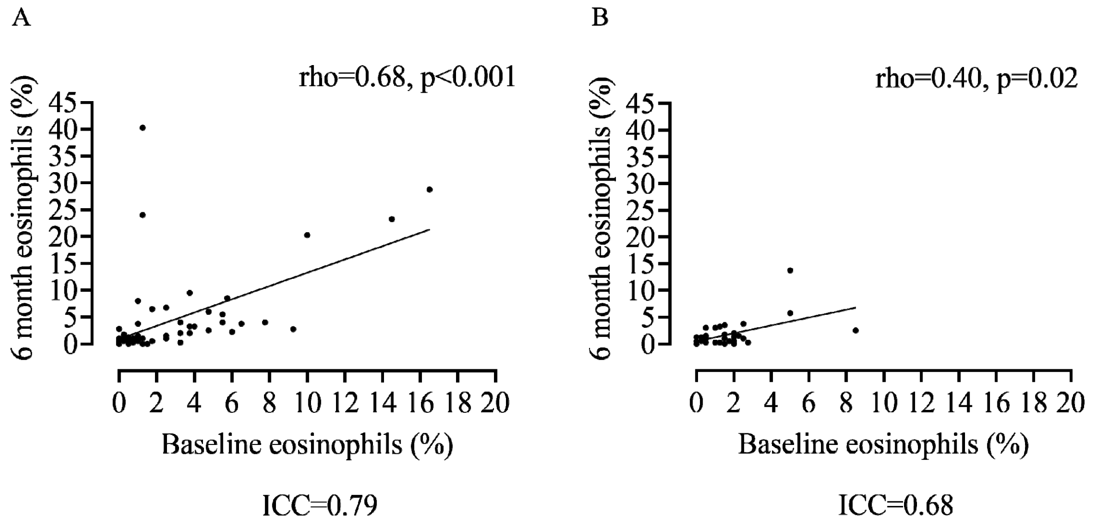

Figure S1. Association between baseline and 6 month measures of sputum eosinophil % for ICS users (A) versus non-users (B). n=66 and 34 respectively

Data normalised via Log(x+1) for calculation of ICC

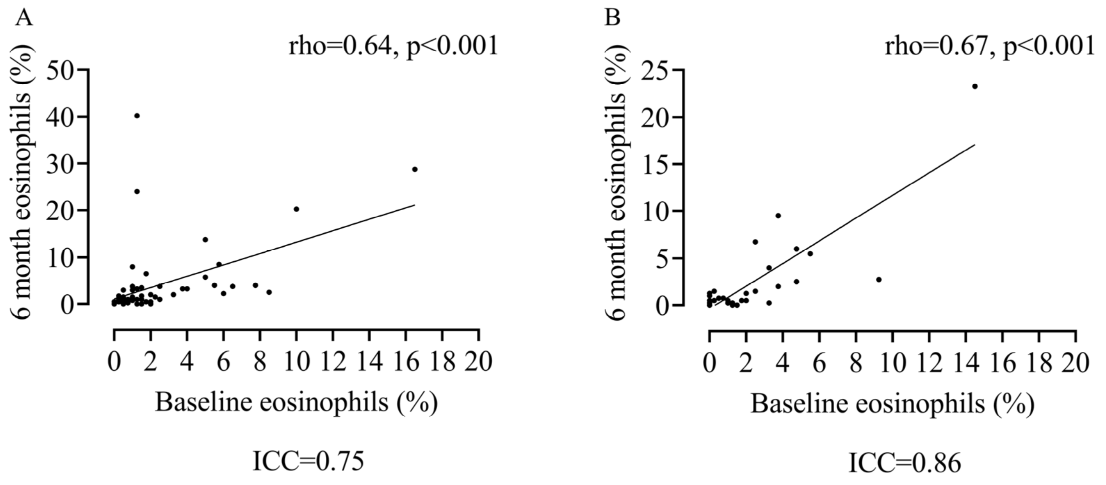

Figure S2. Association between baseline and 6 month measures of sputum eosinophil % for those with no exacerbations between visits (A) versus those with ≥1 (B). n=64 and 32 respectively

Data normalised via Log(x+1) for calculation of ICC

Tables

Table S1. Baseline demographics for separate groups defined by baseline eosinophil %; Eosinophil^{LOW} Eosinophil^{INT} and Eosinophil^{HIGH} : Summaries are presented as percentages or Mean (SD) as appropriate (n=43, 35, 22 for Eosinophil^{LOW,} Eosinophil^{INT} and Eosinophil^{HIGH} respectively[#]). Post-hoc analyses for chi-squared tests were performed, with a Bonferroni correction.

Characteristic	**Eosinophil^{LOW} n=43**	**Eosinophil^{INT} n=35**	**Eosinophil^{HIGH} n=22**	p-value
Gender n (Female/Male)	17/26	14/21	6/16	0.40
Age	64.3 (8.0)	67.1 (6.4)	63.7 (7.7)	0.15
Smoking status (Current %)	37.2	37.1	59.1	0.18
Pack years	**35.0 (13.8)**	****52.5 (22.8)**	$^{+}$**40.2 (13.6)**	**<0.01**
BMI (kg/m^2)	27.6 (5.2)	27.8 (5.7)	28.3 (3.8)	0.87
Exacerbations (1 year period)	0.97 (1.3)	0.71 (0.94)	1.0 (1.3)	0.86
0 (%)	51.4	51.7	52.9	0.24
1 (%)	22.9	31.1	17.6	0.70
≥2 (%)	25.7	17.2	29.5	0.63
Exacerbations (between visits)	0.60 (1.2)	0.38 (0.65)	0.59 (0.96)	0.72
Post-BD FEV_1 (L)	1.74 (0.56)	1.69 (0.49)	1.92 (0.49)	0.26
Post-BD FEV_1 (%)	64.0 (17.0)	63.3 (15.7)	62.6 (21.1)	0.96
GOLD Category (%)				
1	16.3	14.3	18.2	0.92
2	65.1	62.9	68.2	0.92
3	18.6	22.8	13.6	0.69
4	0.0	0.0	0.0	-
mMRC	3.0 [0.0-4.0]	3.0 [0.0-4.0]	4.0 [0.0-4.0]	0.41
CAT	18.6 (8.3)	19.1 (6.7)	21.4 (6.6)	0.67
SGRQ-C (Total)	46.8 (16.9)	47.9 (19.1)	50.0 (22.0)	0.88
Atopy (%)	13.2	6.3	10.0	0.63

Chronic bronchitis (%)	79.2	70.8	73.3	0.80
ICS Use (%)	**69.8**	**48.6**	+**86.4**	**0.01**
LABA+LAMA+ICS (%)	53.5	40.0	72.7	-
LABA+LAMA (%)	2.3	11.4	9.1	-
ICS only (%)	2.3	0.0	4.5	-
LABA only (%)	2.3	0.0	0.0	-
LAMA only (%)	16.3	20.0	0.0	-
No inhaled medication (%)	9.3	11.4	1.5	-

[#]The following data were missing; 20 retrospective exacerbation history, 4 exacerbation history between visits, 10 atopy categorisation, 37 chronic bronchitis categorisation, 25 mMRC questionnaires, 29 CAT questionnaires and 37 SGRQ's.

[**], $p<0.01$ compared to Eos^{LOW}

[+], $p<0.05$ compared to Eos^{INT}

BD, bronchodilator; BMI, body mass index; CAT, COPD assessment test; DCC, differential cell count; FEV_1, forced expiratory volume in 1 second; FVC, forced vital capacity; GOLD, Global Initiative for Chronic Obstructive Lung Disease; ICS, inhaled corticosteroids; LABA, long acting beta agonist; LAMA, long acting muscarinic antagonist; mMRC, modified medical re-search council questionnaire; SGRQ, St George's respiratory questionnaire.

Table S2. Baseline bacterial qPCR results for common respiratory pathogens measured in sputum, for separate groups defined by baseline eosinophil %; Eosinophil^{LOW} Eosinophil^{INT} and Eosinophil^{HIGH} : n=13, 12 and 9 respectively. Summaries are presented as percentages or median [range] as appropriate. *Haemophilus influenzae*, *H.influenzae; Moraxella catarrhalis, M.catarrhalis;* potentially pathogenic microorganism, PPM; *Pseudomonas aeruginosa*, *P.aeruginosa*; quantitative polymerase chain reaction, qPCR; reverse, R; *Streptococcus pneumoniae*, *S.pneumoniae.*

**Baseline sputum characteristic**	**Eosinophil^{LOW} n=13**	**Eosinophil^{INT} n=12**	**Eosinophil^{HIGH} n=9**	**p-value**
**Total PPM Load (genome copies/ml)**	8.12E+05 [0.00E+00-9.25E+07]	2.03E+05 [2.48E+02-1.58E+08]	8.73E+03 [0.00E+00-5.12E+06]	0.25
***H.influenzae* Load (genome copies/ml)**	**2.67E+04 [0.00E+00-9.05E+07]**	**4.12E+03 [0.00E+00-1.58E+08]**	***2.75E+02 [0.00E+00-1.63E+04]**	**0.03**
***S.pneumoniae* Load (genome copies/ml)**	2.42E+05 [0.00E+00-2.02E+06]	2.81E+04 [0.00-1.25E+06]	8.53E+03 [0.00E+00-5.12E+06]	0.99
***M.catarrhalis* Load (genome copies/ml)**	0.00E+00 [0.00E+00-5.63E+02]	0.00E+00 [0.00E+00-1.34E+04]	0.00E+00 [0.00E+00-3.27E+02]	0.30
***P.aeruginosa* Load (genome copies/ml)**	0.00E+00 [0.00E+00-5.88E+06]	0.00E+00 [0.00E+00-0.00E+00]	0.00E+00 [0.00E+00-0.00E+00]	0.46
**No colonisation (% of patients)**	53.8	66.7	100.0	0.06
***H.Influenzae* (% of patients)**	38.5	33.3	0.0	0.11
***S.Pneumoniae* (% of patients)**	0.0	0.0	0.0	n/a
***M.Catarrhalis* (% of patients)**	0.0	8.3	0.0	0.39
***P.Aeruginosa* (% of patients)**	7.7	0.0	0.0	0.44
**>1 ppm (% of patients)**	0.0	8.3	0.0	0.39

*, p<0.05 compared to Eos^{LOW}

Table S3. Details of qPCR targets and the lower limits of detection for qPCR detection of different PPMs.

Target species	Gene target	Primers	Sequence (5'-3')	Probe sequence with Reporter and Quencher	Lower limit of detection (genome copies/ml)
H.influenzae	P4 Lipoprotein gene	HI-F	CCgggTgCggTAgAATTTAATAA	6FAM-ACAGCCACAACGGTAAAGTGTTCTACG-DB	$8.70x10^{1}$
		HI-R	CTgATTTTTCAgTgCTgTCTTTgC		
S.pneumoniae	Spn9082 gene fragment	SP-F	AgTCgTTCCAAggTAACAAgTCT	ROX-TACATGTAGGAAACTATTTTCCTCACAAA-BHQ2	$1.38x10^{3}$
		SP-R	ACCAACTCgACCACCTCTTT		
M.catarrhalis	CopB outer membrane protein gene	MC-F	gTgAgTgCCgCCAAgACAA	6JOE-TGCTTTTGCAGCTGTTAGCCAGCCTAA-BHQ1	$1.76x10^{2}$
		MC-R	TgTATCgCCTgCCAAgACAA		
P.aeruginosa	gyrB gene	PA-F	TCCAAGTTTAAGGTGGTAGGCTG	FAM-AGG TAA ATC CGG GGT TTC AAG GCC-TAMRA	6.00
		PA-R	ACCACTTCGTCAATCTAAAAGACGA		

Forward, F; *Haemophilus influenzae*, *H.influenzae; Moraxella catarrhalis, M.catarrhalis; Pseudomonas aeruginosa*, *P.aeruginosa*; quantitative polymerase chain reaction, qPCR; reverse, R; *Streptococcus pneumoniae*, *S.pneumoniae.*

Table S4. Baseline demographics for separate groups defined by change in eosinophil % between baseline and 6 months using a threshold of 3% sputum eosinophils; Eosinophil Persistently >3%, Eosinophil Intermittently >3% , Eosinophil <3%: Summaries are presented as percentages or Mean (SD) as appropriate (n=15, 18, 67 for Eosinophil Persistently >3% , Eosinophil Intermittently >3% , Eosinophil <3% respectively[#]).

Characteristic	Eosinophil Persistently >3% n=15	Eosinophil Intermittently >3% n=18	Eosinophil <3% n=67	p-value
Gender (Female/Male)	2/13	9/11	23/44	0.14
Age	61.1 (8.17)	65.8 (6.8)	65.2 (7.6)	0.81
Smoking status (Current %)	53.3	44.4	38.8	0.57
Pack years	41.5 (14.2)	45.8 (21.7)	41.5 (19.2)	0.69
BMI (kg/m^2)	27.4 (3.5)	29.9 (4.1)	27.4 (5.5)	0.16
Exacerbations (1 year period)	0.9 (1.2)	1.2 (1.3)	0.8 (1.2)	0.54
0 (%)	58.3	35.7	55.6	0.38
1 (%)	16.7	28.6	24.1	0.77
≥2 (%)	25.0	35.7	20.3	0.48
Exacerbations (between visits)	0.5 (1.1)	0.3 (0.6)	0.6 (1.1)	0.67
Post-BD FEV_1 (L)	1.9 (0.5)	1.7 (0.4)	1.8 (0.5)	0.53
Post-BD FEV_1 (%)	63.3 (14.5)	62.2 (22.2)	63.8 (16.8)	0.94
Δ Post-BD FEV_1 (L)	-0.0 (0.2)	0.0 (0.2)	-0.0 (0.2)	0.71
Δ Post-BD FEV_1 (%)	-0.1 (5.9)	0.0 (6.5)	-1.9 (8.4)	0.54
GOLD Category (%)				
1	13.3	16.7	16.4	0.95
2	73.3	66.7	62.7	0.73
3	13.4	16.6	20.9	0.77
4	0.0	0.0	0.0	-
mMRC	4.0 [0-4]	3.0 [1.0-4.0]	3.0 [0.0-4.0]	0.52
CAT	21.7 (7.6)	17.7 (4.8)	19.2 (7.9)	0.47
SGRQ-C (Total)	53.8 (24.6)	43.2 (16.2)	47.5 (17.6)	0.50

Atopy (%)	15.4	5.9	10.0	0.69
Chronic bronchitis (%)	70.0	75.0	75.6	0.93
ICS Use (%)	86.7	66.7	61.2	0.17

#The following data were missing; 20 retrospective exacerbation history, 4 exacerbation history between visits, 10 atopy categorisation, 37 chronic bronchitis categorisation, 25 mMRC questionnaires, 29 CAT questionnaires and 37 SGRQ's, 3 Δ Post-BD FEV_1 (L).

BD, bronchodilator; BMI, body mass index; CAT, COPD assessment test; DCC, differential cell count; FEV_1, forced expiratory volume in 1 second; GOLD, Global Initiative for Chronic Obstructive Lung Disease; ICS, inhaled corticosteroids; LABA, long acting beta agonist; LAMA, long acting muscarinic antagonist; mMRC, modified medical re-search council questionnaire; SGRQ, St George's respiratory questionnaire; Δ, change between baseline and 6 month visit.

References

1. McCulloch, E.; Lucas, C.; Ramage, G.; Williams, C. Improved early diagnosis of Pseudomonas aeruginosa by real-time PCR to prevent chronic colonisation in a paediatric cystic fibrosis population. *J Cyst Fibros* **2011**, *10*, 21-24, doi:10.1016/j.jcf.2010.09.001.
2. Garcha, D.S.; Thurston, S.J.; Patel, A.R.; Mackay, A.J.; Goldring, J.J.; Donaldson, G.C.; McHugh, T.D.; Wedzicha, J.A. Changes in prevalence and load of airway bacteria using quantitative PCR in stable and exacerbated COPD. *Thorax* **2012**, *67*, 1075-1080, doi:10.1136/thoraxjnl-2012-201924.
3. Beech, A.; Lea, S.; Li, J.; Jackson, N.; Mulvanny, A.; Singh, D. Airway Bacteria Quantification Using Polymerase Chain Reaction Combined with Neutrophil and Eosinophil Counts Identifies Distinct COPD Endotypes. *Biomedicines* **2021**, *9*, doi:10.3390/biomedicines9101337.
